# Association of poorly controlled HbA_1c_ with increased risk of progression to end-stage kidney disease and all-cause mortality in patients with diabetes and chronic kidney disease

**DOI:** 10.1371/journal.pone.0274605

**Published:** 2022-09-26

**Authors:** Sheng-Jen Chen, Hsiu-Yin Chiang, Pei-Shan Chen, Shih-Ni Chang, Sheng-Hsuan Chen, Min-Yen Wu, Hung-Chieh Yeh, I-Wen Ting, Hsiu-Chen Tsai, Pei-Chun Chen, Chin-Chi Kuo

**Affiliations:** 1 Department of Education, China Medical University Hospital and College of Medicine, China Medical University, Taichung, Taiwan; 2 Big Data Center, China Medical University Hospital, Taichung, Taiwan; 3 Division of Nephrology, Department of Internal Medicine, China Medical University Hospital and College of Medicine, China Medical University, Taichung, Taiwan; 4 Department of Internal Medicine, AKI-CARE (Clinical Advancement, Research and Education) Center, China Medical University Hospital and College of Medicine, China Medical University, Taichung, Taiwan; 5 Department of Public Health, China Medical University, Taichung, Taiwan; Faculty of Medicine, Saint-Joseph University, LEBANON

## Abstract

Glycosylated hemoglobin (HbA1c) targets for patients with chronic kidney disease (CKD) and type 2 diabetes remain controversial. To evaluate whether baseline HbA_1c_ and HbA_1c_ trajectories are associated with the risk of end-stage kidney disease (ESKD) and all-cause mortality, we recruited adult patients with CKD and type 2 diabetes from a “Pre-ESKD Program” at a medical center in Taiwan from 2003 to 2017. Group-based trajectory modeling was performed to identify distinct patient groups that contained patients with similar longitudinal HbA1c patterns. Cox proportional hazard models were used to estimate hazard ratios (HRs) of ESKD and mortality associated with baseline HbA_1c_ levels and HbA_1c_ trajectories. In the analysis related to baseline HbA_1c_ (*n* = 4543), the adjusted HRs [95% confidence interval (CI)] of all-cause mortality were 1.06 (0.95–1.18) and 1.25 (95% CI, 1.07–1.46) in patients with an HbA_1c_ level of 7%–9% (53–75 mmol/mol) and >9% (>75 mmol/mol), respectively, as compared with those with an HbA1c level < 7% (<53 mmol/mol). In the trajectory analysis *(n* = 2692), three distinct longitudinal HbA_1c_ trajectories were identified: nearly optimal (55.9%), moderate to stable (34.2%), and poor control (9.9%). Compared with the “nearly optimal” HbA_1c_ trajectory group, the “moderate-to-stable” group did not have significantly higher mortality, but the “poorly controlled” group had 35% higher risk of mortality (adjusted HR = 1.35, 95% CI = 1.06–1.71). Neither baseline levels of HbA_1c_ nor trajectories were associated with ESKD risk. In conclusion, in patients with CKD and type 2 diabetes, poor glycemic control was associated with an elevated risk of mortality but not associated with a risk of progression to ESKD.

## Introduction

Diabetic nephropathy is a leading cause of end-stage kidney disease (ESKD) worldwide and accounts for a considerable proportion of the global ESKD incidence, including in Singapore (66.4%), the United States (46.9%), Taiwan (46.2%), Japan (42.5%), Canada (37.7%), and the United Kingdom (26.5%) [1]. For patients with coexisting type 2 diabetes and chronic kidney disease (CKD), optimal glycemic control targets have been explored in diverse populations. Currie et al. [2] reported a U-shaped association between all-cause mortality and glycosylated hemoglobin (HbA_1c_) levels in patients with diabetes. In the Action to Control Cardiovascular Risk in Diabetes (ACCORD) [3] trial, the risk of all-cause mortality among patients with CKD stage 1–3 was higher in the intensive therapy group (median HbA_1c_ of approximately 6.5%, 48 mmol/mol in the 12th month of follow-up) than in the standard therapy group (median HbA_1c_ of approximately 7.6%, 60 mmol/mol in the 12th month of follow-up). Shurraw et al. [4] revealed a U-shaped association between the risk of all-cause mortality and a baseline HbA_1c_ level of <6.5% (48 mmol/mol) or > 8.0% (64 mmol/mol). Another study on baseline HbA_1c_ revealed that compared with patients who had CKD stage 3 or 4 and a baseline HbA_1c_ level < 6.0%, patients with CKD stage 3 or 4 and a baseline HbA_1c_ level > 9.0% (75 mmol/mol) had higher risk of ESKD (rather than all-cause mortality). However, the ESKD risk was lower in patients with CKD stage 5 [5]. The latest KDIGO (Kidney Disease: Improving Global Outcomes) guidelines suggest that the acceptable HbA_1c_ target ranges from 6.5%–8.0% (48–64 mmol/mol) [6]; however, this consensus on a glycemic control target was mainly based on clinical trials in which patients with preserved kidney function (i.e., those with an estimated glomerular filtration rate [eGFR] of ≧60 mL/min/1.73 m^2^) were selected [7–9]. Whether the study findings can be generalized to patients with coexisting diabetes and advanced CKD in real-world settings deserves attention [3, 10, 11].

None of the aforementioned studies have explored the prognostic role of the longitudinal trajectory of HbA_1c_ level in patients with type 2 diabetes and CKD; such an exploration could aid in optimal glycemic control threshold estimation. Although a single value of HbA_1c_ could reflect the average blood glucose level over a period of up to 3 months, its representativeness of longer-term glycemic control is insufficient, and thus, up to four annual HbA_1c_ measurements have been suggested [6]. Although the KDIGO Work Group noted the potential for more stringent glycemic control to improve clinical outcomes in terms of all-cause mortality, cardiovascular death, and CKD progression [6], more robust evidence is required to verify whether stringent glycemic control can modify the disease course of patients with type 2 diabetes and CKD. In this study, we used a 15-year single-center longitudinal database to systematically investigate the association of both baseline HbA_1c_ levels and HbA_1c_ trajectories with the risk of progression to ESKD and all-cause mortality in patients with type 2 diabetes and CKD.

## Materials and methods

### Study population

In 2002, Taiwan’s National Health Insurance launched the Project of Integrated Care of CKD and, since 2007, the project’s focus has been CKD stages 3b–5 [12]. This pre-end-stage kidney disease (ESKD) program was a multidisciplinary approach to the design of individualized care plans for a wide range of patients with CKD. Patients with eGFR < 45 mL/min/1.73 m^2^ (i.e., CKD stage 3b–5), or with eGFR ≥ 45 mL/min/1.73 m^2^ (i.e., CKD stage 1–3a) with evident proteinuria (urine protein and creatinine ratio ≥ 1000 mg/gm) were eligible to participate in the Pre-ESKD Program. The objective was to meet the therapeutic goals listed in the guidelines of the National Kidney Foundation Kidney Disease Outcomes Quality Initiative [13]. China Medical University Hospital (CMUH), a tertiary medical center located in Central Taiwan, joined the Pre-ESKD program in 2003. Consecutive patients with CKD who were willing to participate were prospectively enrolled. The CMUH pre-ESKD program currently includes more than 11 000 participants and has an overall retention rate of 90%. CKD diagnoses are based on the working diagnoses of nephrologists or the criteria outlined in the aforementioned initiative’s guidelines [13]. Patients in CKD stages 3b, 4, and 5 were, respectively, followed up at 12, 8, and 4 weeks, or as necessary. Biochemical markers of renal injury including serum creatinine, eGFR, and the spot urine protein–creatinine ratio (PCR) were measured at intervals of no more than 12 weeks. Detailed information on the Pre-ESKD Program has been provided previously [14, 15]. Throughout the manuscript, we use the phrase Pre-ESKD (end-stage kidney disease) program to refer to this multidisciplinary care program.

**Fig 1:**
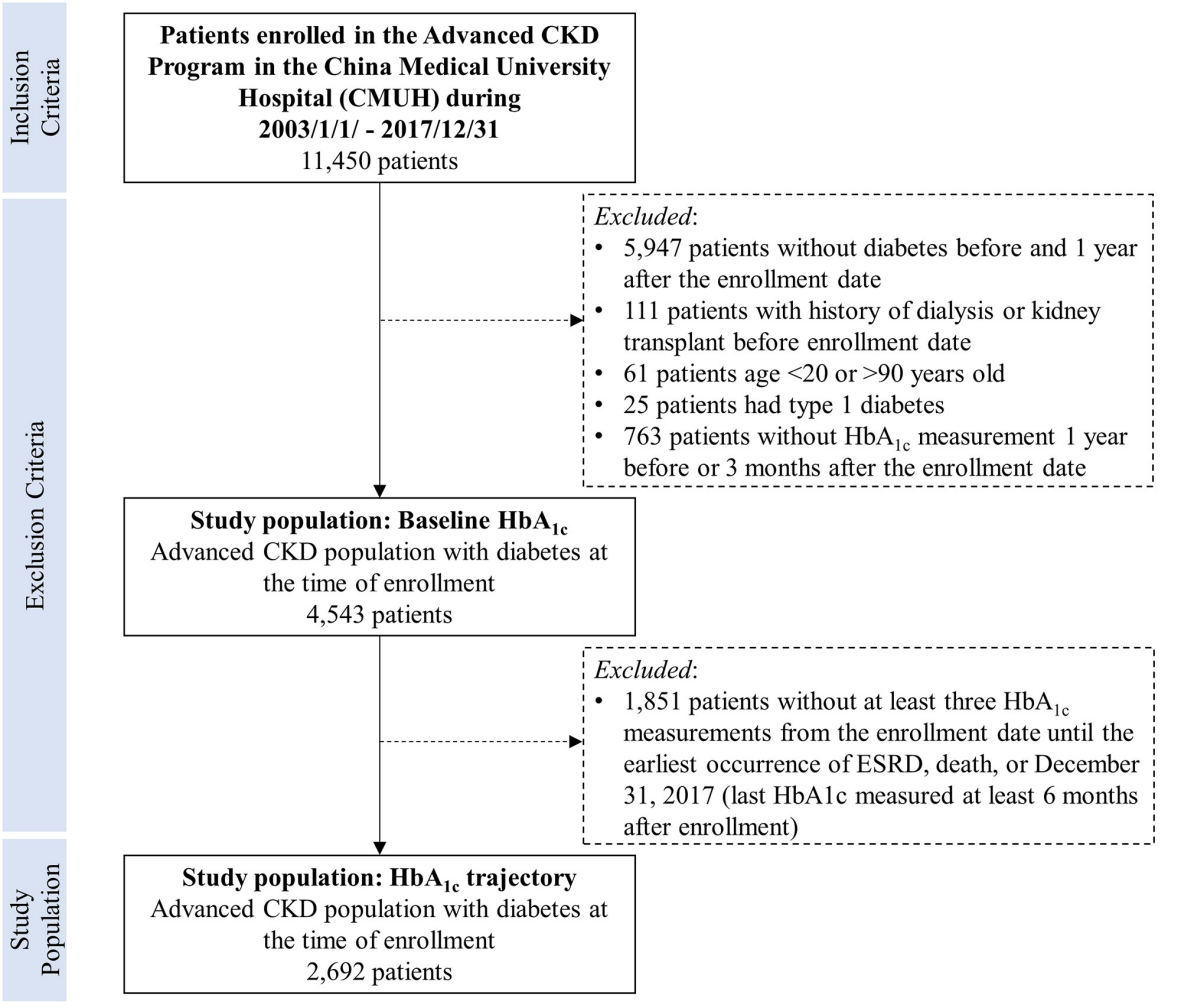
Selection of the study population.

The index date was defined as the date of first enrollment in the Pre-ESKD program. We first identified patients with a diagnosis of diabetes based on the International Classification of Diseases, 9th and 10th revision Clinical Modification (ICD-9-CM 250 or ICD-10-CM E08-E11, E13) codes and prescriptions of antidiabetic agents before the index date as well as during an additional 1-year inclusion window following the index date. The exclusion criteria included (1) age < 20 years or > 90 years, (2) having a history of dialysis or kidney transplant before the index date, (3) having type 1 diabetes, and (4) not having a recorded baseline HbA_1c_ value. Baseline HbA_1c_ was defined as the HbA_1c_ value recorded 1 year before or 3 months after the index date; the measurement closest to the index date was used. Patients with type 1 diabetes were identified from certificates of catastrophic illness issued by the National Health Insurance Administration, Ministry of Health and Welfare of Taiwan. Because we wanted to observe longitudinal HbA1c patterns, only patients with at least three measurements of HbA1c were included in the trajectory analysis. Patients included in the HbA_1c_ trajectory analysis was had to have had at least a 6-month follow-up and a last HbA1c measurement at least 6 months after the index date (Fig 1). Consequently, 4543 patients were included in the baseline HbA_1c_ analysis and 2692 patients were included in the trajectory analysis (Fig 1).

### Measurement of HbA_1c_

All HbA_1c_ levels were measured at the central laboratory of CMUH. Before September 2013, HbA_1c_ was measured using Tosoh’s Automated Glycohemoglobin Analyzer HLC-723G7 (Tosoh G7; Tosoh Corporation, Minato-Ku, Tokyo, Japan). Two-point calibration was performed using a standard HbA_1c_ sample after every device power-up. The analyzer could distinguish between labile A_1c_ and stable HbA_1c_, indicating a minimal risk of measurement error. Calculation of HbA_1c_ levels was based on the ratio of the stable HbA_1c_ fraction chromatographic area to that of total glycosylated hemoglobin, and the HbA1_c_ ratio of each result was automatically adjusted using the calibration equation [16]. From September 2013 onward, the HbA_1c_ measurement protocol was switched; Premier Hb9210 (Trinity Biotech Plc., Wicklow, Ireland.) was used thereafter. Premier Hb9210 uses boronate-affinity high-performance liquid chromatography to detect all types of the presented glycosylated Hb species. The final HbA_1c_ results are determined from a simple peak area fraction.

According to prespecified HbA1c values from the literature and the latest American Diabetes Association practice guidelines, we divided the patients into three groups: those with a baseline HbA_1c_ level < 7% (<53 mmol/mol), 7%–9% (53–75 mmol/mol), and >9% (>75 mmol/mol); [4, 5, 17]. In the trajectory analysis, we used all available HbA_1c_ measurements collected during follow-up for each patient to determine the patient subgroups with similar patterns in longitudinal HbA_1c_.

### Other covariables

Sociodemographic variables, including age, sex, education level, smoking status, and alcohol consumption, were collected through a questionnaire during enrollment. Smoking status and alcohol consumption status were categorized as never, former, and current [18]. Body mass index (BMI) was calculated as weight in kilograms divided by height in meters squared, and the latest measurements obtained within ±2 years of the index date were used in the analysis. Baseline levels of all biochemical variables were determined using the latest measurements obtained within 90 days to 1 year of the index date. eGFR was calculated using the CKD epidemiology collaboration equation: [eGFR = 141 × min (S-Cre/κ, 1)^α^ × max(S-Cre/κ, 1)^−1.209^ × 0.993^age^ × 1.018 [if patient is female] × 1.159 [if Black], where S-Cre is serum creatinine, κ is 0.7 for female patients and 0.9 for male patients, and α is −0.329 for female patients and −0.411 for male patients] [19]. The baseline eGFR of each patient was determined using their serum creatinine level, and patients were assigned to corresponding CKD stages based on the following cutoff values: > 90, 60–89.9, 30–59.9, 15–29.9, and < 15 mL/min/1.73 m^2^. CKD stage was then determined on the basis of the following cutoff values for eGFR: >90 (stage 1), 60–89.9 (stage 2), 30–59.9 (stage 3), 15–29.9 (stage 4), and <15 (stage 5) mL/min/1.73 m^2^. Missing values of the pooled urine protein–creatinine ratio (uPCR) were estimated from the urine albumin–creatine ratio (uACR) by using the following formula: ln(uACR) = 1.32 × ln(uPCR) − 2.64 [20]. Data on comorbidities and medication use were collected by searching the electronic health records within 1 year before the index date. Hypertension was defined as the presence of related diagnosis codes (ICD-9 codes 401–405 and ICD-10 codes I10–I15) or the prescription of an antihypertensive agent. Cardiovascular disease (CVD) included coronary artery disease, myocardial infarction, stroke, or heart failure (ICD-9 codes 394.9, 396, 410–414, 422.9, 424.0–424.2, 428.0, 428.9, 429.2, 430–438, and ICD-10 codes G45-G46, I11.0, I13.0, I13.2, I20-I25, I50, I60-I63, I69).

### Outcomes and follow-up

Survival status and date of death was ascertained through data linkage with the National Death Registry of Taiwan. To minimize bias, we created a proxy outcome for progression to ESKD—a doubling of serum creatinine (S-Cre) concentration—in the main analysis to balance the risk of dialysis among the three baseline HbA1c groups. Progression to ESKD was defined as the initiation of peritoneal dialysis, hemodialysis, kidney transplantation, and doubling of S-Cre compared with the baseline. For each study participant, the follow-up period was from the index date until the earliest occurrence of ESKD, loss to follow-up, death, or December 31, 2017 whichever occurred first.

### Statistical analyses

Continuous variables were expressed as a median and interquartile range (IQR), and the differences in continuous variables among the groups were determined using the Wilcoxon rank sum test. Categorical variables were expressed as percentages, and the differences in categorical variables among the HbA_1c_ categories were examined using a chi-squared test. *P* values for trends were calculated using Spearman’s correlation for continuous variables and the Cochran–Armitage trend test for categorical variables.

A semiparametric group-based trajectory model (GBTM) was used to characterize the distinct trajectories of HbA_1c_ during the follow-up period. The PROC TRAJ macro, developed using the SAS software package, fits a semiparametric mixture model to longitudinal data by using the maximum likelihood method [21–23]. GBTM is a useful approach for trajectory characterization when the number of potential subgroups and trajectory shapes of each subgroup are still unclear, and the Bayesian information criterion was employed to assess model fit by balancing model complexity. We empirically processed 2- and 3-group solutions and focused on the 3-group solution eventually after considering the sample size and facilitation of meaningful clinical interpretations. Missing values of sociodemographic variables were imputed using multiple imputation under the “missing at random” assumption. Associations of baseline HbA_1c_ and HbA_1c_ trajectories with the risk for ESKD and all-cause mortality were assessed using Cox proportional hazard models with age as the time scale. The subdistribution hazard model developed by Fine and Gray was fitted for ESKD; it accounted for competing risks of death without ESKD. We constructed three models with increasing levels of covariate adjustment. Model 1 was adjusted for sex, body mass index, smoking status, alcohol consumption, and education. Model 2 was further adjusted for systolic blood pressure, cardiovascular disease, primary etiologies of CKD, baseline medication use (contrast, nonsteroidal anti-inflammatory drugs, oral antidiabetic agents, insulin, angiotensin-converting enzyme inhibitors, angiotensin receptor blockers, diuretics, and epoetin), triglyceride level, and low-density lipoprotein cholesterol level. Model 3 was adjusted for all variables in Model 2 and baseline hemoglobin, eGFR, and pooled uPCR.

The dose–response relationship of baseline HbA_1c_ levels with all-cause mortality and ESKD risk was characterized using a restricted cubic spline in the Cox regression analysis with knots at the 10th, 50th, and 90th percentiles of the overall distribution of HbA_1c_ levels. We further performed exploratory subgroup analyses to evaluate potential effect modifications in the fully adjusted model according to age (< 65 vs. ≥ 65 years), sex, BMI category (< 25 vs. ≥ 25 kg/m^2^), smoking status, alcohol consumption, CKD stage (1–2 vs. 3–5), hypertension, and CVD. All statistical analyses were performed using SAS (version 9.4; SAS Institute Inc., Cary, NC, USA) and R (version 3.6.0; R Foundation for Statistical Computing, Vienna, Austria).

### Ethics statement

All methods used in this study were performed in accordance with the relevant guidelines and regulations. The study was approved by the Big Data Center of CMUH and the Research Ethical Committee/Institutional Review Board of China Medical University Hospital (CMUH105-REC3-068); the need to obtain written informed consent for the present study was waived by the Research Ethical Committee of CMUH.

## Results

### Characteristics of study subjects by baseline HbA_1c_ levels

Among the 4543 subjects included in the baseline HbA1c analysis, the median age at enrollment was 67.6 years (IQR: 59.2–75.7), the median HbA_1c_ level was 7.1% (IQR: 6.30–8.20), and the median eGFR was 26.5 mL/min/1.73 m^2^ (IQR: 13.8–43.8; Table 1). The median follow-up duration was 1.6 (IQR: 0.7–3.0) years for the development of ESKD and 3.8 (IQR: 1.9–6.3) years for all-cause mortality. At baseline, of all patients, 89.74% had a urine PCR value of ≥150 mg/g and 85.7% had a urine ACR value of ≥30 mg/g. Patients with a higher baseline HbA_1c_ level were younger and tended to have longer follow-up durations of ESKD and higher BMI (Table 1). In addition, patients with a higher HbA_1c_ level had a higher eGFR. Correspondingly, the proportion of CKD stage 5 and phosphorus and albumin levels were significantly lower in patients with a higher HbA1c level than in those with a lower HbA_1c_ level. Overall, 25.7% and 51.2% of our population were treated with an angiotensin-converting enzyme inhibitor (ACEi) and angiotensin II receptor blocker (ARB), respectively. The proportion of patients with progression to ESKD was significantly lower in the group with baseline HbA_1c_ of 7–9% (53–75 mmol/mol) whereas all-cause mortality was comparable in the three groups “Table 1”.

**Table 1:** Demographic and clinical characteristics of the study population by baseline HbA_1c_ categories.

Characteristics ^a^			Baseline HbA_1c_				
	N	Total (n = 4543)	<7%	7–9%	>9%	P-value ^b^	P for trend ^b^
			(<53 mmol/mol) (n = 2126)	(53–75 mmol/mol) (n = 1798)	(>75 mmol/mol) (n = 619)		
**Demographic information, median (IQR)**							
Age at entry (year)	4543	67.6 (59.2, 75.7)	68.7 (60.2, 76.7)	67.4 (59.4, 75.3)	63.9 (54.6, 73.4)	< 0.001	< 0.001
Male, n (%)	4543	2507 (55.2)	1202 (56.5)	950 (52.8)	355 (57.4)	0.034	0.514
Education level (year), n (%)	4543					0.650	-
< 9		1191 (26.2)	560 (26.3)	478 (26.6)	153 (24.7)		
9 ≤ ~ <12		1991 (43.8)	921 (43.3)	799 (44.4)	271 (43.8)		
12 ≤ ~ <16		968 (21.3)	448 (21.1)	375 (20.9)	145 (23.4)		
16+		393 (8.7)	197 (9.3)	146 (8.1)	50 (8.1)		
Follow up duration of ESKD (year)	4543	1.6 (0.7, 3.0)	1.5 (0.6, 3.0)	1.8 (0.9, 3.1)	1.9 (0.9, 3.0)	< .001	< .001
Follow up duration of mortality (year)	4543	3.8 (1.9, 6.3)	3.4 (1.6, 6.0)	4.2 (2.2, 6.4)	4.0 (2.2, 6.3)	< .001	< .001
Body mass index (kg/m^2^) ^c^	4504	25.1 (22.8, 27.9)	24.9 (22.5, 27.6)	25.2 (23.0, 28.0)	25.4 (23.1, 28.8)	< 0.001	< 0.001
Systolic blood pressure (mmHg)	4512	135 (127, 150)	135 (127, 150)	135 (127, 150)	135 (127, 150)	0.855	0.992
Diastolic blood pressure (mmHg)	4512	79 (70, 81)	78 (69, 80)	80 (70, 82)	80 (70, 85)	< 0.001	< 0.001
**Behavioral, n (%)**							
Smoking status	4543					0.080	-
Never		3699 (81.4)	1743 (82.0)	1471 (81.8)	485 (78.4)		
Former		381 (8.4)	187 (8.8)	138 (7.7)	56 (9.1)		
Current		463 (10.2)	196 (9.2)	189 (10.5)	78 (12.6)		
Alcohol consumption	4543					0.111	-
Never		4116 (90.6)	1931 (90.8)	1640 (91.2)	545 (88.1)		
Former		274 (6.0)	129 (6.1)	95 (5.3)	50 (8.1)		
Current		153 (3.4)	66 (3.1)	63 (3.5)	24 (3.9)		
**Baseline comorbidities** ^d^**, n (%)**							
Hypertension	4533	3377 (74.5)	1571 (74.1)	1361 (75.9)	445 (72.0)	0.136	0.760
Cardiovascular disease	4533	1823 (40.2)	829 (39.1)	756 (42.1)	238 (38.5)	0.098	0.562
Primary etiologies of CKD	4535					< 0.001	-
Renal Parenchymal Diseases		418 (9.2)	281 (13.3)	102 (5.7)	35 (5.7)		
Systemic Disease		4040 (89.1)	1783 (84.2)	1678 (93.3)	579 (93.5)		
Obstructive Nephropathy and Urinary Tract Diseases		39 (0.9)	28 (1.3)	9 (0.5)	2 (0.3)		
Other		38 (0.8)	26 (1.2)	9 (0.5)	3 (0.5)		
CKD stage	4538					< 0.001	-
Stage 1–2		477(10.5)	157 (7.4)	226 (12.6)	94 (15.22)		
Stage 3		1611(35.5)	700 (33.0)	671 (37.3)	240 (38.8)		
Stage 4		1257(27.7)	571 (26.9)	517 (28.8)	169 (27.3)		
Stage 5		1193(26.3)	695 (32.7)	383 (21.3)	115 (18.6)		
**Baseline medication profiles** ^d^**, n (%)**							
Nonsteroidal anti-inflammatory drugs	4480	1134 (25.3)	528 (25.3)	445 (25.0)	161 (26.3)	0.833	0.737
Contrast	4480	680 (15.2)	317 (15.2)	259 (14.6)	104 (17.0)	0.364	0.508
Anti-diabetic agents							
Oral antidiabetic agents	4480	2963 (66.1)	1280 (61.2)	1244 (70.0)	439 (71.6)	< 0.001	< 0.001
Insulin	4480	1791 (40.0)	678 (32.4)	765 (43.1)	348 (56.8)	< 0.001	< 0.001
Anti-hypertensive agents							
Angiotensin-converting enzyme inhibitors	4480	1152 (25.7)	510 (24.4)	469 (26.4)	173 (28.2)	0.115	0.038
Angiotensin II receptor blockers	4480	2293 (51.2)	1013 (48.5)	977 (55.0)	303 (49.4)	< 0.001	0.051
Diuretics	4480	2661 (59.4)	1249 (59.8)	1043 (58.7)	369 (60.2)	0.726	0.907
β blockers	4480	1942 (43.4)	925 (44.3)	761 (42.8)	256 (41.8)	0.465	0.218
Anti-lipid agents							
Statin	4480	1443 (32.2)	577 (27.6)	640 (36.0)	226 (36.9)	< 0.001	< 0.001
Fibrate	4480	384 (8.6)	145 (6.9)	164 (9.2)	75 (12.2)	< 0.001	< 0.001
Anti-platelet agents							
Aspirin, Ticlopidine, Clopidogrel	4480	621 (13.9)	270 (12.9)	268 (15.1)	83 (13.5)	0.148	0.276
Dipyridamole	4480	288 (6.4)	141 (6.8)	109 (6.1)	38 (6.2)	0.719	0.486
Epoetin	4480	563 (12.6)	359 (17.2)	159 (9.0)	45 (7.3)	< 0.001	< 0.001
**Baseline biochemical profiles** ^e^**, median (IQR)**							
Glucose AC (mg/dL)	4178	127 (105, 159)	114 (99, 132)	140 (112, 170)	185 (138, 236)	< 0.001	< 0.001
HbA_1c_ (%)	4543	7.10 (6.30, 8.20)	6.30 (5.90, 6.60)	7.70 (7.30, 8.30)	10.20 (9.60, 11.10)	< 0.001	< 0.001
HbA_1c_ (mmol/mol)	4543	54 (45, 66)	45 (41, 49)	61 (56, 67)	88 (81, 98)	< 0.001	< 0.001
Serum creatinine (mg/dL)	4540	2.16 (1.45, 3.74)	2.40 (1.57, 4.35)	2.07 (1.39, 3.23)	1.90 (1.35, 3.09)	< 0.001	< 0.001
eGFR (mL/min/1.73m^2^)	4540	26.5 (13.8, 43.8)	23.2 (11.2, 40.5)	28.9 (16.1, 46.2)	31.5 (18.3, 50.1)	< 0.001	< 0.001
Uric acid (mg/dL)	4115	7.40 (6.20, 8.80)	7.40 (6.20, 8.80)	7.40 (6.20, 8.80)	7.30 (5.90, 8.50)	0.099	0.112
Blood urea nitrogen (mg/dL)	4267	35.0 (23.0, 55.0)	37.0 (24.0, 60.0)	34.0 (22.0, 51.0)	31.0 (21.0, 49.0)	< 0.001	< 0.001
Sodium (mmol/L)	4102	138 (135, 140)	138 (136, 140)	137 (135, 140)	137 (134, 139)	< 0.001	< 0.001
Potassium (mmol/L)	4295	4.30 (3.90, 4.70)	4.30 (3.90, 4.80)	4.30 (3.90, 4.70)	4.20 (3.80, 4.60)	< 0.001	< 0.001
Calcium (mg/dL)	3697	8.80 (8.30, 9.20)	8.70 (8.30, 9.20)	8.90 (8.40, 9.20)	8.80 (8.40, 9.20)	< .0001	< 0.001
Phosphorus (mg/dL)	3512	4.20 (3.70, 4.90)	4.30 (3.70, 5.10)	4.20 (3.70, 4.80)	4.10 (3.60, 4.80)	< 0.001	< 0.001
Albumin (g/dL)	3976	3.90 (3.40, 4.20)	3.90 (3.35, 4.30)	3.90 (3.40, 4.20)	3.80 (3.30, 4.10)	0.007	0.019
Hemoglobin (g/dL)	3726	10.7 (9.3, 12.2)	10.3 (9.1, 11.9)	10.9 (9.6, 12.4)	11.1 (9.7, 12.9)	< 0.001	< 0.001
Total cholesterol (mg/dL)	4107	176 (148, 211)	171 (144, 205)	179 (152, 212)	188 (158, 225)	< 0.001	< 0.001
Triglyceride (mg/dL)	4322	142 (99, 212)	129 (91, 190)	149 (104, 222)	174 (117, 282)	< 0.001	< 0.001
LDL-C (mg/dL)	3462	97 (75, 122)	95 (73, 120)	97 (76, 123)	100 (77, 130)	0.007	0.002
HDL-C (mg/dL)	2824	38.9 (32.8, 47.1)	38.8 (32.7, 47.3)	39.0 (32.8, 47.3)	38.7 (33.2, 46.0)	0.798	0.924
Urine PCR (mg/g)	3353	1499 (361, 4308)	1554 (341, 4157)	1373 (349, 4279)	1614 (482, 5006)	0.036	0.201
> = 150 mg/g, n (%)		3009 (89.74)	1431 (88.28)	1159 (89.57)	419 (95.66)	< 0.001	< 0.001
Urine ACR (mg/g)	2119	421 (65, 2229)	469 (60, 2187)	393 (67, 2194)	441 (74, 2440)	0.363	0.217
> = 30 mg/g, n (%)	1816	1816 (85.7)	743 (82.83)	781 (86.2)	292 (92.41)	< 0.001	< 0.001
Urine Routine Protein (UA) upon 2+, n(%)		2105 (56.69)	1002 (56.83)	799 (55.29)	304 (60.20)	0.158	0.475
Albuminuria (defined as urine PCR > = 150 mg/g or urine	3939	3853 (97.82)	1764 (97.14)	1530 (98.01)	559 (99.47)	0.003	< 0.001
ACR > = 30 mg/g or UA upon 2+), n (%)							
Pooled urine PCR (mg/g)	2468	808 (226, 2,701)	926 (212, 2,848)	713 (234, 2,512)	797 (291, 2,439)	0.828	0.708
**Outcome, n (%)**							
ESKD	4543	2053 (45.2)	954 (44.9)	804 (44.7)	295 (47.7)	0.024	0.655
All-cause mortality	4543	1698 (37.4)	810 (38.1)	652 (36.3)	236 (38.1)	0.455	0.639

^a.^ Categorical variables are presented as frequency (%) and continuous variables are presented as median (IQR).

^b^ P-values are calculated by chi-square test for categorical variables and Wilcoxon rank sum test for continuous variables. Spearman’s correlation was adopted for analyzing P value for trend of continuous variables, and Cochran-Armitage trend test were applied to calculating P-value for trend of categorical variables.

^c^ Baseline body mass index were the latest measurements that were obtained within -2 years to +2 years of the index date.

^d^ Baseline comorbidities and medication profiles that occurred within 1 year prior to the index date.

^e^ Baseline biochemical profiles were the latest measurements that were obtained within -1 year to +90 days of the index date.

CKD: chronic kidney disease, LDL-C: low-density lipoprotein cholesterol, HDL-C: high-density lipoprotein cholesterol, PCR: protein/creatinine ratio, ACR: albumin/creatinine ratio, ESKD: end-stage kidney disease.

### Characteristics of participants by HbA_1c_ trajectories

**Fig 2:**
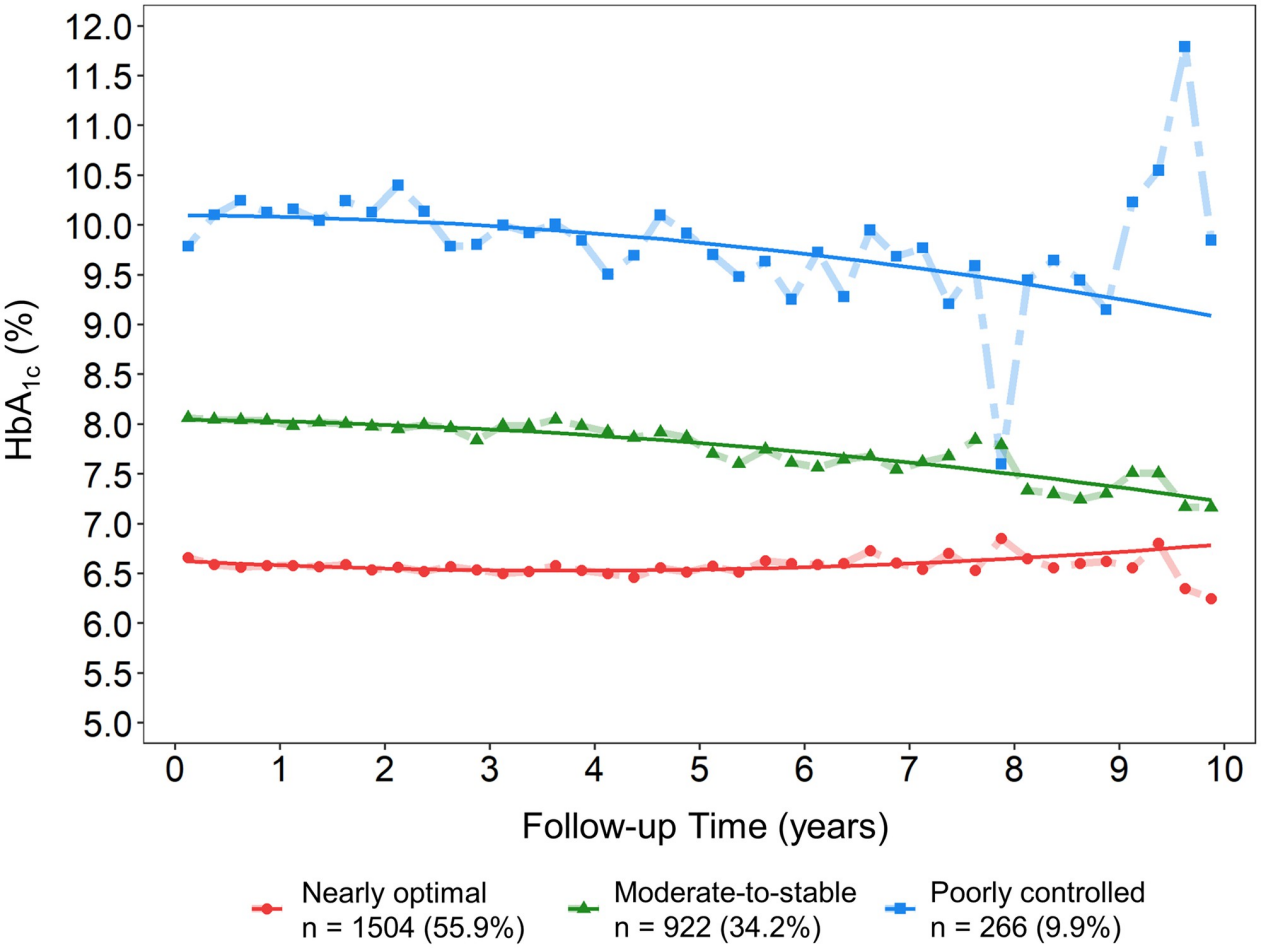
HbA_1c_ trajectories by group-based trajectory modeling as per the three-trajectory solution. The solid lines indicate the mean estimated trajectories; the points represent the mean observed trajectories.

Overall, 2692 patients were enrolled in the trajectory analysis, and the median number of HbA_1c_ measurements was 8 (IQR: 5–14) per patient during the study period. The median follow-up duration was 2.6 (IQR: 1.6–4.0) years for the development of ESKD and 4.4 (IQR: 2.7–6.5) years for all-cause mortality. Three distinct longitudinal HbA_1c_ trajectories were identified by the GBTM: nearly optimal (55.9%), moderate-to-stable (34.2%), and poorly controlled (9.9%) (Fig 2). The HbA_1c_ trajectory of the “nearly-optimal” group was stably below a HbA_1c_ level of 7% (53 mmol/mol), whereas the “moderate-to-stable” and “poorly controlled” groups had HbA_1c_ trajectories that fluctuated at approximately 8% (64 mmol/mol) and 10% (86 mmol/mol), respectively. Both the “moderate-to-stable” and “poorly controlled” groups had downward trends in HbA_1c_ levels during the follow-up, particularly in the case of the “poorly controlled” group. Compared with the “nearly optimal” and “moderate-to-stable” groups, patients in the “poorly controlled” group—similar to those with baseline HbA_1c_ levels of > 9% (> 75 mmol/mol)—were younger and tended to have longer follow-up for progression to ESKD and a higher BMI. Those patients were also less likely to have baseline CKD stage 4–5 with a corresponding higher eGFR at baseline (S1 Table). Deviating slightly from the observations made in the baseline analysis, the proportion of patients with progression to ESKD was slightly higher in the “poorly controlled” group than in the other groups, whereas all-cause mortality was comparable among the three groups (S1 Table).

### ESKD risk and all-cause mortality based on baseline HbA_1c_ and HbA_1c_ trajectory

**Fig 3:**
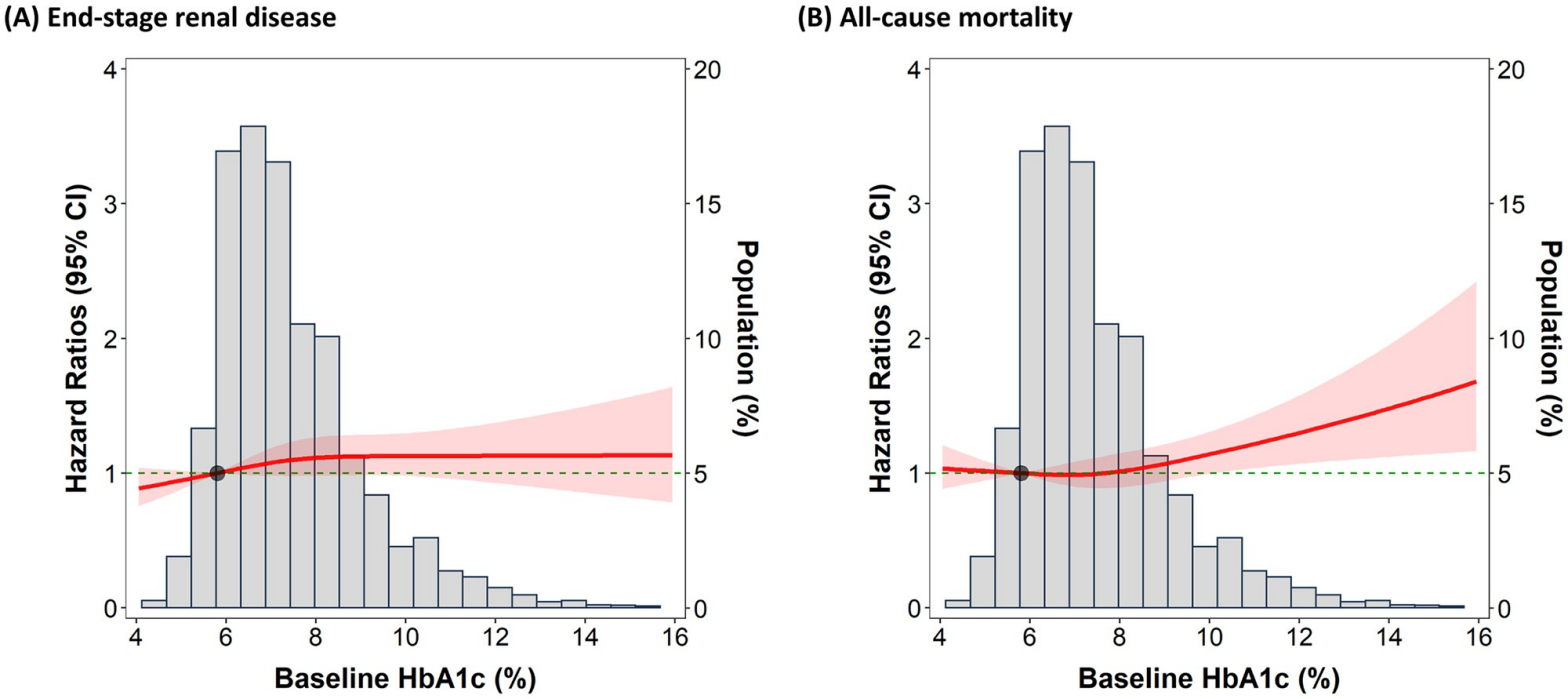
Dose-response plot of the baseline HbA1c and adjusted hazard ratios for (A) progression to end-stage kidney disease and (B) all-cause mortality according to baseline HbA_1c_ (%). Solid lines represent adjusted hazard ratios based on restricted cubic splines for baseline HbA_1c_, with knots at the 10th, 50th, and 90th percentiles. Shaded areas represent the upper and lower 95% confidence intervals. The reference was set at the 10th percentile of HbA_1c_ levels. Variables adjusted are the same as that shown in Model 3 presented in Table 2. Missing values were imputed by multiple imputation.

**Fig 4:**
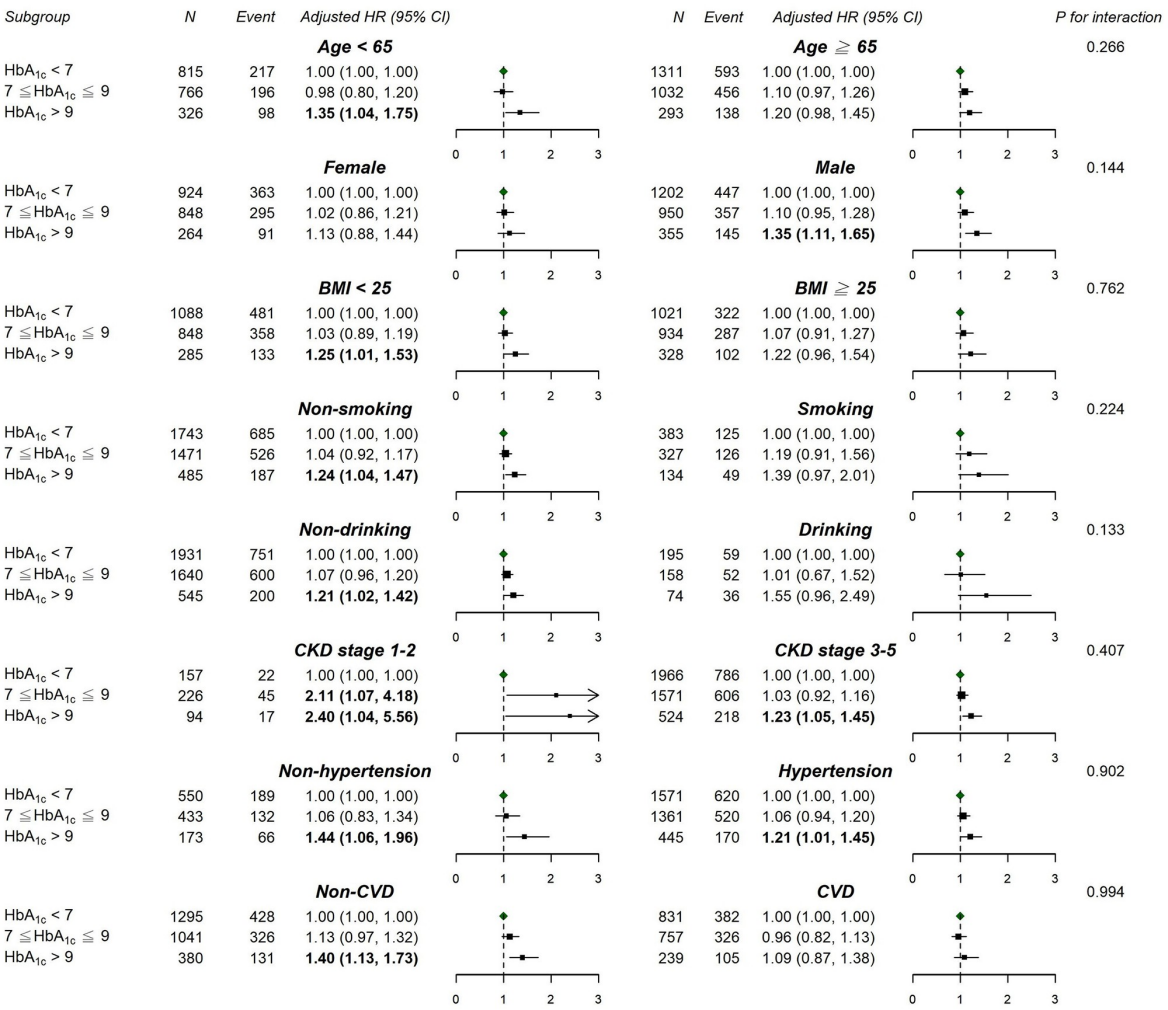
Subgroup analysis of the hazard ratios (95% confidence interval) of all-cause mortality associated with baseline HbA_1c_ groups. BMI: body mass index, CKD: chronic kidney disease, CVD: cardiovascular disease.

The analysis of baseline HbA_1c_, revealed that 2053 ESKD events and 1698 deaths occurred over a total 9888 and 19 253 person-years of follow-up, respectively. The incidence and HR of developing ESKD and all-cause mortality are revealed in Table 2. In the unadjusted model, a modest inverse association was found between the baseline HbA1c category and risk of ESKD (*P* for trend = 0.028); the HR (95% CI) for a baseline HbA1c level of 7%–9% (53–75 mmol/mol) and >9% (> 75 mmol/mol) versus an HbA_1c_ level of <7% (53 mmol/mol) was 0.92 (0.83–1.01) and 0.87 (0.76–1.002), respectively. The inverse association remained, although attenuated, after controlling for demographics, smoking, and alcohol consumption in model 1 and additionally after controlling for systolic blood pressure, cardiovascular disease, lipid levels, primary etiologies of CKD, and medication use in model 2. However, in model 3, in which baseline hemoglobin, eGFR, and pooled uPCR were additionally controlled, the inverse association between the baseline HbA_1c_ level and risk of progression to ESKD was not found. We did not discover a significant association between the baseline HbA_1c_ level and all-cause mortality in the unadjusted model and adjusted models 1 and 2. However, a positive association was found in model 3 (*P* for trend = 0.009); the HR (95% CI) for a baseline HbA_1c_ of >9% (>75 mmol/mol) versus <7% (53 mmol/mol) was 1.25 (1.07–1.46). The dose–response curve between baseline HbA_1c_ levels and the risk of all-cause mortality in model 3 showed a monotonic relationship (*P* = 0.02; Fig 3B), but such a relationship did not appear between baseline HbA_1c_ and risk of ESKD (Fig 3A). An exploratory subgroup analysis revealed that the associations between the baseline HbA_1c_ level and risk of all-cause mortality were consistent in patient subgroups stratified in accordance with *in priori* selected variables (Fig 4). Generally, the association between a high baseline HbA_1c_ level and all-cause mortality was stronger in patients free of advanced CKD, hypertension, and CVD.

**Table 2:** Hazard ratios (95% confidence interval) of progression to end-stage kidney disease (ESKD) and all-cause mortality associated with baseline HbA_1c_ and HbA_1c_ trajectory groups.

						Model 1	Model 2	Model 3
	N	cases	Person-years	Incidence^a^	Crude HR	Adjusted HR	Adjusted HR	Adjusted HR
					(95% CI)	(95% CI)	(95% CI)	(95% CI)
**Progression to ESKD** ^b^^,^ ^c^^,^ ^d^								
**Baseline HbA**_**1c**_ (%)								
< 7	2126	954	4390.44	217.29	1.00 (Ref)	1.00 (Ref)	1.00 (Ref)	1.00 (Ref)
7–9	1798	804	4069.44	197.57	0.92 (0.83, 1.01)	0.92 (0.84, 1.02)	0.94 (0.85, 1.04)	1.08 (0.97, 1.2)
> 9	619	295	1427.73	206.62	0.87 (0.76, 1.002)	0.88 (0.76, 1.01)	0.89 (0.77, 1.03)	1.11 (0.94, 1.3)
*P* for trend					0.028	0.035	0.096	0.139
**HbA**_**1c**_ **trajectory**								
Nearly optimal	1504	682	4360.09	156.42	1.00 (Ref)	1.00 (Ref)	1.00 (Ref)	1.00 (Ref)
Moderate-to-stable	922	406	2943.69	137.92	0.96 (0.86, 1.08)	0.97 (0.86, 1.09)	0.94 (0.83, 1.06)	1.03 (0.92, 1.16)
Poorly controlled	266	119	812.29	146.5	1.01 (0.84, 1.20)	1.01 (0.84, 1.21)	0.97 (0.81, 1.15)	1.13 (0.94, 1.35)
*P* for trend					0.791	0.848	0.445	0.234
**All-cause mortality** ^c^^,^ ^d^								
**Baseline HbA**_**1c**_ (%)								
< 7	2126	810	8584.97	94.35	1.00 (Ref)	1.00 (Ref)	1.00 (Ref)	1.00 (Ref)
7–9	1798	652	7941.58	82.1	0.91 (0.82, 1.01)	0.92 (0.83, 1.02)	0.93 (0.84, 1.04)	1.06 (0.95, 1.18)
> 9	619	236	2726.49	86.56	1.04 (0.90, 1.21)	1.05 (0.91, 1.22)	1.03 (0.88, 1.19)	1.25 (1.07, 1.46)
*P* for trend					0.781	0.921	0.821	0.009
**HbA**_**1c**_ **trajectory**								
Nearly optimal	1504	444	6845.93	64.86	1.00 (Ref)	1.00 (Ref)	1.00 (Ref)	1.00 (Ref)
Moderate-to-stable	922	268	4655.37	57.57	0.98 (0.84, 1.14)	0.97 (0.84, 1.13)	0.96 (0.82, 1.12)	1.07 (0.91, 1.25)
Poorly controlled	266	87	1244.28	69.92	1.26 (0.99, 1.59)	1.29 (1.02, 1.63)	1.16 (0.91, 1.48)	1.35 (1.06, 1.71)
*P* for trend					0.223	0.183	0.513	0.031

^a^ Incidence = No. of incident progression to ESKD or mortality cases/ person-years*1000.

^b.^ Cox proportional hazards analysis with the competing risk of death by subdistribution hazard model was performed for the outcome of progression to ESKD.

^c^ Model 1: Adjusted for sex, body mass index, smoking status, alcohol consumption, education (Baseline HbA_1c_: n = 4543; HbA_1c_ trajectory: n = 2692). Model 2: Further adjusted for systolic blood pressure, cardiovascular disease, primary etiologies of chronic kidney disease, baseline medication (contrast, nonsteroidal anti-inflammatory drugs, oral antidiabetic agents, insulin, angiotensin-converting enzyme inhibitors, angiotensin receptor blockers, diuretics, epoetin), triglyceride and low-density lipoprotein cholesterol. Model 3: Further adjusted for baseline hemoglobin, estimated glomerular filtration rate, and pooled urine protein/creatinine ratio.

^d^ Age was used as time scale.

In analysis of HbA_1c_ trajectories, 1157 ESKD events and 799 deaths occurred over a total 8116 and 12 745 person-years of follow-up, respectively. No significant association was discovered between the HbA1c trajectory categories and risk of developing ESKD in the unadjusted model or any adjusted model (Table 2). In model 3, the adjusted HR (95% CI) of progression to ESKD was 1.03 (0.92–1.16) for the “moderate-to-stable” HbA_1c_ group and 1.13 (0.94–1.35) for the “poor control” group as compared with the “nearly optimal” group (Table 2). However, the HbA1c trajectory categories were associated with all-cause mortality. In model 3, the “poor control” group had 35% higher risk of mortality (6%–71%) than the “nearly optimal” group (Table 2).

## Discussion

Our findings revealed that a high HbA_1c_ level at the time of Pre-ESKD Program enrollment and a poorly controlled HbA_1c_ trajectory over the follow-up period were associated with increased risk of all-cause mortality in patients with type 2 diabetes and CKD. Despite emerging evidence endorsing the relaxation of HbA_1c_ as a goal for older patients with multiple comorbidities including CKD, maintaining the longitudinal HbA_1c_ level at <9% (75 mmol/mol) remains vital for improving patients’ overall survival. The null associations of progression to ESKD with a high baseline HbA_1c_ level and a “poorly controlled” HbA_1c_ trajectory should be interpreted cautiously, because the risk of ESKD associated with HbA_1c_ levels may have been modified by the differential erythrocyte lifespan between early and advanced of CKD.

The relatively linear dose–response relationship between the baseline HbA_1c_ level and risk of all-cause mortality was inconsistent with the findings of a study by Shurraw et al. [4], who found a U-shaped relationship between baseline HbA_1c_ and all-cause mortality in patients with CKD stage 3 and 4. In further analyses of Shurraw et al. in which CKD stages 3 and 4 were stratified separately, the magnitude of the increased risk of ESKD associated with poor glycemic control—single baseline HbA_1c_ level >9% (>75 mmol/mol) as opposed to <7% (<53 mmol/mol)—was greater among patients with CKD stage 3 than among those with CKD stage 4 [4]. This minor discrepancy between our study and that of Shurraw et al. is likely due to differences in the study population and statistical approaches. First, the present study further included patients with CKD stage 5 and patients with CKD stages 1–3a with evident proteinuria. Second, our study was based on a well-interoperated dataset incorporating a single institution’s electronic medical records and the national Pre-ESKD Program, meaning that our confounding control was better because variables such as smoking status, alcohol consumption, hemoglobin level, proteinuria, lipid profile, and medication use were available [4]. Conversely, most studies have demonstrated a positive association between baseline HbA_1c_ and the risk of progression to ESKD [4, 5]. On the basis of overarching findings across studies including our own, we can conclude that avoiding an HbA_1c_ level of >9% (>75 mmol/mol) is likely to benefit patients with type 2 diabetes and CKD, even when the disease stage is advanced. Notably, we did not observe better kidney prognosis and mortality outcomes in patients with an HbA_1c_ level of <7% (<53 mmol/mol) compared with those having an HbA_1c_ level of 7%–9% (53–75 mmol/mol). However, whether the therapeutic goal of an HbA_1c_ level >7%–7.5% should be relaxed is beyond the scope of this study and requires clinical and research consensus concerning the definition of intensive glycemic control for patients with diabetes and CKD (ADA 2022) [24].

Few empirical studies have explored the prognostic role of longitudinal trends in HbA_1c_ in patients with type 2 diabetes and CKD. A study of 770 patients with type 2 diabetes and CKD demonstrated that a “moderate increase” HbA_1c_ trajectory was associated with increased risk of CKD progression compared with a “near-optimal stable” trajectory [25]. The kidney function of that study population was relatively well preserved (median eGFR = 84.8 mL/min/1.73 m^2^). In addition, instead of the development of ESKD, CKD progression was defined by a decline in CKD stage with a ≥25% reduction of baseline eGFR [25]. The consistently observed association between a poor glycemic control trajectory and increased risks of ESKD and mortality in patients with diabetes and CKD highlights the importance of taking proactive measures to prevent hyperglycemic states over the course of CKD care. An integrated CKD care program should target diabetic patients with poor long-term glycemic control, particularly those with early CKD.

This study has several limitations. First, this was a retrospective cohort study and we could not derive causal inferences from its results. Second, selection bias due to correlation between the HbA_1c_ level and CKD stage should be considered. Briefly, patients with more advanced CKD would have shorter red blood cell survival, leading to a relatively low HbA_1c_ level even in a similar glycemic milieu. Patients with CKD in the lower stratum of baseline HbA_1c_ were more likely to have a more advanced CKD stage and therefore progress more rapidly to ESKD. To minimize this bias, we created a proxy outcome for progression to ESKD—a doubling of the S-Cre concentration—in the main analysis; this balanced the risk of dialysis among the three baseline HbA_1c_ groups. We also restricted our analysis of patients with CKD stage 3 and found that a baseline HbA_1c_ level of >9% (>75 mmol/mol) was significantly associated with increased risk of progression to ESKD [aHR 1.35 (95% CI, 1.04–1.75)] but not increased risk of mortality [aHR 1.05 (95% CI, 0.78–1.41)], as compared with the <7% (53 mmol/mol) group. This observation provides a complimentary perspective to our main findings and supports the hypothesis that an HbA_1c_ level > 9%, is associated with increased risk of both ESKD and mortality in patients with CKD. The effect of poorly controlled HbA_1c_ may be modified by an inherited propensity toward outcomes of interest, which was introduced by the differential erythrocyte lifespan across CKD stages. Third, the possibility of residual confounding—such as a lack of access to detailed dietary information and compliance with medication—could not be completely excluded. Fourth, the follow-up duration may have been insufficient to observe the development of ESKD in patients with CKD stages 1–3. To minimize the impact of the potentially insufficient follow-up, we also used doubling of S-Cre as a surrogate endpoint to define the progression of CKD to ESKD.

## Conclusion

In individuals with CKD and type 2 diabetes, maintaining the HbA_1c_ level < 9% (<75 mmol/mol) remains crucial for halting CKD progression and reducing the mortality risk. Patients in the early stages of CKD were particularly vulnerable to the negative effects of chronic hyperglycemia and accelerated progression to ESKD. Whether the development and integration of a glycemic optimization protocol into the existing CKD program for patients with diabetes and CKD can help lower the CKD-related healthcare burden requires clinical trial validation and thus warrants further study.

## Supporting information

S1 Table Demographic and clinical characteristics of the study population by the longitudinal HbA_1c_ trajectories. (DOCX)

S2 Table Hazard ratios (95% confidence interval) of 30% decline of estimated glomerular filtration rate (eGFR), doubling serum creatinine, progression to end-stage kidney disease (ESKD), and all-cause mortality associated with baseline HbA_1c_ groups. (DOCX)
