# Supplementary material for: Association of poorly controlled HbA1c with increased risk of progression to end-stage kidney disease and all-cause mortality in patients with diabetes and chronic kidney disease
Source: PLoS One. 2022 Sep 26;17(9):e0274605. doi: 10.1371/journal.pone.0274605 (PMC9512200; doi:10.1371/journal.pone.0274605)
Supplement: S1 Table — (DOCX) [file pone.0274605.s001.docx]

**S1 Table.** Demographic and clinical characteristics of the study population by the longitudinal HbA_1c_ trajectories.

| **Characteristics ^a^** |  |  | **HbA_1c_ trajectory** | | |  |  |
| --- | --- | --- | --- | --- | --- | --- | --- |
|  | N | Total (n = 2692) | Nearly optimal (n = 1504) | Moderate-to-stable (n = 922) | Poorly controlled (n = 266) | P-value ^b^ | P for trend ^b^ |
| **Demographic information, median (IQR)** |  |  |  |  |  |  |  |
| Age at entry (year) | 2692 (100) | 68.2 (59.8, 75.8) | 69.4 (61.3, 76.7) | 66.8 (58.6, 75.0) | 65.2 (56.9, 73.4) | <.001 | <.001 |
| Male, n (%) | 2692 (100) | 1477 (54.9) | 851 (56.6) | 483 (52.4) | 143 (53.8) | 0.122 | 0.095 |
| Education level (year), n (%) | 2692 (100) |  |  |  |  | 0.682 | - |
| < 9 |  | 687 (25.5) | 388 (25.8) | 228 (24.7) | 71 (26.7) |  |  |
| 9 ≤ ~ <12 |  | 1186 (44.1) | 651 (43.3) | 416 (45.1) | 119 (44.7) |  |  |
| 12 ≤ ~ <16 |  | 565 (21.0) | 311 (20.7) | 197 (21.4) | 57 (21.4) |  |  |
| 16+ |  | 254 (9.4) | 154 (10.2) | 81 (8.8) | 19 (7.1) |  |  |
| No. of HbA1c records | 2692 (100) | 8.0 (5.0, 14.0) | 7.0 (4.0, 13.0) | 9.0 (5.0, 15.0) | 8.0 (5.0, 14.0) | <.001 | <.001 |
| Follow up duration of ESKD (year) | 2692 (100) | 2.6 (1.6, 4.0) | 2.5 (1.5, 3.8) | 2.7 (1.7, 4.3) | 2.6 (1.7, 3.9) | 0.002 | 0.001 |
| Follow up duration of mortality (year) | 2692 (100) | 4.4 (2.7, 6.5) | 4.1 (2.6, 6.3) | 5.0 (2.8, 6.5) | 4.3 (2.7, 6.4) | <.001 | <.001 |
| Body mass index (kg/m^2^) ^c^ | 2679 (99.5) | 25.4 (23.2, 28.1) | 25.3 (22.8, 27.7) | 25.5 (23.4, 28.4) | 26.3 (23.7, 29.7) | <.001 | <.001 |
| Systolic blood pressure (mmHg) | 2688 (99.9) | 132.0 (125.0, 145.0) | 133.0 (125.0, 145.0) | 133.0 (126.0, 145.0) | 130.0 (123.0, 146.0) | 0.647 | 0.989 |
| Diastolic blood pressure (mmHg) | 2688 (99.9) | 77.0 (69.0, 80.0) | 76.0 (68.0, 80.0) | 77.0 (69.0, 80.0) | 80.0 (70.0, 84.0) | 0.001 | 0.006 |
| **Behavioral, n (%)** |  |  |  |  |  |  |  |
| Smoking status | 2692 (100) |  |  |  |  |  |  |
| Never |  | 2181 (81) | 1223 (81.3) | 741 (80.4) | 217 (81.6) | 0.094 | - |
| Former |  | 238 (8.8) | 146 (9.7) | 71 (7.7) | 21 (7.9) |  |  |
| Current |  | 273 (10.1) | 135 (9) | 110 (11.9) | 28 (10.5) |  |  |
| Alcohol consumption | 2692 (100) |  |  |  |  |  |  |
| Never |  | 2440 (90.6) | 1350 (89.8) | 845 (91.7) | 245 (92.1) | 0.453 | - |
| Former |  | 163 (6.1) | 100 (6.7) | 51 (5.5) | 12 (4.5) |  |  |
| Current |  | 89 (3.3) | 54 (3.6) | 26 (2.8) | 9 (3.4) |  |  |
| **Baseline comorbidities ^d^, n (%)** |  |  |  |  |  |  |  |
| Hypertension | 2685 (99.7) | 2066 (76.9) | 1154 (76.9) | 710 (77.1) | 202 (76.5) | 0.936 | 0.948 |
| Cardiovascular disease | 2685 (99.7) | 1145 (42.6) | 651 (43.4) | 377 (40.9) | 117 (44.3) | 0.45 | 0.686 |
| Primary etiologies of CKD | 2689 (99.9) |  |  |  |  | < 0.001 | - |
| Renal Parenchymal Diseases |  | 234 (8.7) | 163 (10.9) | 55 (6) | 16 (6) |  |  |
| Systemic Disease |  | 2413 (89.7) | 1307 (87.1) | 857 (93) | 249 (93.6) |  |  |
| Obstructive Nephropathy and Urinary Tract Diseases |  | 21 (0.8) | 18 (1.2) | 3 (0.3) | 0 (0) |  |  |
| Other |  | 21 (0.8) | 13 (0.9) | 7 (0.8) | 1 (0.4) |  |  |
| CKD stage | 2689 (99.9) |  |  |  |  | < 0.001 | - |
| Stage 1-2 |  | 394 (14.7) | 179 (11.9) | 161 (17.5) | 54 (20.3) |  |  |
| Stage 3 |  | 1143 (42.5) | 623 (41.5) | 392 (42.5) | 128 (48.1) |  |  |
| Stage 4 |  | 753 (28) | 424 (28.3) | 263 (28.5) | 66 (24.8) |  |  |
| Stage 5 |  | 399 (14.8) | 275 (18.3) | 106 (11.5) | 18 (6.8) |  |  |
| **Baseline medication profiles ^d^, n (%)** |  |  |  |  |  |  |  |
| Nonsteroidal anti-inflammatory drugs | 2661 (98.8) | 689 (25.9) | 391 (26.4) | 228 (24.9) | 70 (26.7) | 0.754 | 0.748 |
| Contrast | 2661 (98.8) | 395 (14.8) | 215 (14.5) | 130 (14.2) | 50 (19.1) | 0.134 | 0.184 |
| Anti-diabetic agents |  |  |  |  |  |  |  |
| Oral antidiabetic agents | 2661 (98.8) | 1950 (73.3) | 1071 (72.2) | 681 (74.3) | 198 (75.6) | 0.272 | 0.153 |
| Insulin | 2661 (98.8) | 1007 (37.8) | 476 (32.1) | 390 (42.6) | 141 (53.8) | <.001 | <.001 |
| Anti-hypertensive agents |  |  |  |  |  |  |  |
| Angiotensin-converting enzyme inhibitors | 2661 (98.8) | 674 (25.3) | 382 (25.8) | 219 (23.9) | 73 (27.9) | 0.42 | 0.986 |
| Angiotensin II receptor blockers | 2661 (98.8) | 1466 (55.1) | 810 (54.6) | 518 (56.6) | 138 (52.7) | 0.361 | 0.969 |
| Diuretics | 2661 (98.8) | 1438 (54.0) | 798 (53.8) | 479 (52.3) | 161 (61.5) | 0.043 | 0.183 |
| β blockers | 2661 (98.8) | 1168 (43.9) | 670 (45.2) | 387 (42.2) | 111 (42.4) | 0.392 | 0.177 |
| Anti-lipid agents |  |  |  |  |  |  |  |
| Statin | 2661 (98.8) | 971 (36.5) | 487 (32.8) | 374 (40.8) | 110 (42) | <.001 | <.001 |
| Fibrate | 2661 (98.8) | 267 (10.0) | 119 (8) | 111 (12.1) | 37 (14.1) | <.001 | <.001 |
| Anti-platelet agents |  |  |  |  |  |  |  |
| Aspirin, Ticlopidine, Clopidogrel | 2661 (98.8) | 360 (13.5) | 202 (13.6) | 112 (12.2) | 46 (17.6) | 0.094 | 0.435 |
| Dipyridamole | 2661 (98.8) | 171 (6.4) | 91 (6.1) | 68 (7.4) | 12 (4.6) | 0.186 | 0.949 |
| Epoetin | 2661 (98.8) | 182 (6.8) | 123 (8.3) | 48 (5.2) | 11 (4.2) | 0.004 | 0.001 |
| **Baseline biochemical profiles ^e^, median (IQR)** |  |  |  |  |  |  |  |
| Glucose AC (mg/dL) | 2571 (95.5) | 130.0 (108.0, 162.0) | 121.0 (103.0, 142.0) | 145.0 (117.0, 178.0) | 182.0 (134.0, 236.0) | <.001 | <.001 |
| HbA_1c_ (%) | 2692 (100) | 7.2 (6.5, 8.3) | 6.6 (6.1, 7.2) | 8.0 (7.2, 8.8) | 9.5 (8.4, 10.8) | <.001 | <.001 |
| HbA_1c_ (mmol/mol) | 2692 (100) | 55.2 (47.5, 67.2) | 48.6 (43.2, 55.2) | 63.9 (55.4, 72.7) | 80.3 (68.3, 94.5) | <.001 | <.001 |
| Serum creatinine (mg/dL) | 2690 (99.9) | 1.8 (1.3, 2.8) | 1.9 (1.4, 3.0) | 1.8 (1.2, 2.6) | 1.7 (1.2, 2.3) | <.001 | <.001 |
| eGFR (mL/min/1.73m^2^) | 2690 (99.9) | 33.0 (19.6, 51.0) | 31.3 (18.0, 47.9) | 34.0 (21.3, 54.2) | 36.8 (24.9, 56.5) | <.001 | <.001 |
| Uric acid (mg/dL) | 2412 (89.6) | 7.2 (6.0, 8.6) | 7.3 (6.0, 8.5) | 7.2 (6.0, 8.6) | 7.1 (5.8, 8.6) | 0.391 | 0.805 |
| Blood urea nitrogen (mg/dL) | 2460 (91.4) | 30.0 (21.0, 45.0) | 31.0 (21.0, 48.0) | 29.0 (21.0, 42.0) | 28.0 (19.0, 40.0) | 0.006 | 0.001 |
| Sodium (mmol/dL) | 2345 (87.1) | 138.0 (135.0, 140.0) | 138.0 (136.0, 140.0) | 138.0 (135.0, 140.0) | 137.0 (135.0, 139.0) | <.001 | <.001 |
| Potassium (mmol/dL) | 2491 (92.5) | 4.3 (3.9, 4.7) | 4.3 (3.9, 4.7) | 4.3 (3.9, 4.7) | 4.1 (3.9, 4.5) | 0.003 | 0.078 |
| Calcium (mg/dL) | 2018 (75.0) | 8.9 (8.5, 9.3) | 8.9 (8.5, 9.3) | 9.0 (8.6, 9.3) | 8.9 (8.5, 9.3) | 0.008 | 0.01 |
| Phosphorus (mg/dL) | 1900 (70.6) | 4.1 (3.6, 4.6) | 4.1 (3.6, 4.7) | 4.0 (3.6, 4.5) | 4.0 (3.6, 4.6) | 0.144 | 0.095 |
| Albumin (g/dL) | 2251 (83.6) | 4.0 (3.6, 4.3) | 4.0 (3.6, 4.3) | 4.0 (3.6, 4.3) | 4.0 (3.5, 4.2) | 0.09 | 0.146 |
| Hemoglobin (g/dL) | 2085 (77.5) | 11.1 (9.8, 12.7) | 10.9 (9.6, 12.5) | 11.3 (10.0, 12.9) | 11.7 (10.1, 13.2) | <.001 | <.001 |
| Total cholesterol (mg/dL) | 2507 (93.1) | 175.0 (148.5, 207.0) | 171.0 (145.0, 202.0) | 179.0 (154.0, 212.0) | 179.0 (155.0, 213.0) | <.001 | <.001 |
| Triglyceride (mg/dL) | 2589 (96.2) | 145.0 (101.0, 217.0) | 134.0 (95.0, 194.5) | 161.0 (107.0, 241.8) | 167.0 (119.5, 254.8) | <.001 | <.001 |
| LDL-C (mg/dL) | 2186 (81.2) | 96.2 (75.1, 119.7) | 96.0 (74.6, 118.6) | 96.3 (75.4, 120.8) | 96.1 (78.1, 122.0) | 0.438 | 0.205 |
| HDL-C (mg/dL) | 1847 (68.6) | 38.9 (33.2, 46.8) | 38.7 (32.6, 47.3) | 39.0 (33.7, 46.0) | 39.5 (34.2, 47.2) | 0.488 | 0.282 |
| Urine PCR (mg/g) | 1943 (72.2) | 967.4 (272.4, 2855.8) | 984.3 (252.8, 2859.8) | 931.5 (281.0, 2825.7) | 978.7 (335.9, 2843.6) | 0.734 | 0.468 |
| >=150 mg/g, n(%) | 1943 (72.2) | 1687 (86.8) | 936 (84.7) | 571 (88.5) | 180 (93.3) | 0.204 | <.001 |
| Urine ACR (mg/g) | 1510 (56.1) | 245.0 (53.2, 1506.9) | 236.0 (48.1, 1514.8) | 245.0 (59.2, 1555.7) | 267.4 (55.3, 1303.1) | 0.818 | 0.56 |
| >=30 mg/g, n(%) | 1510 (56.1) | 1269 (84.0) | 678 (81.7) | 456 (86.7) | 135 (87.7) | 0.051 | 0.009 |
| Urine Routine Protein (UA) upon 2+, n(%) | 2153 (80.0) | 1043 (48.4) | 586 (48.1) | 342 (47.4) | 115 (53.7) | 0.188 | 0.322 |
| Albuminuria (defined as urine PCR >=150 mg/g or urine  ACR >=30 mg/g or UA upon 2+), n (%) | 2295 (85.3) | 2230 (97.2) | 1216 (96.1) | 776 (98.1) | 238 (99.6) | 0.001 | < 0.001 |
| Pooled urine PCR (mg/g) | 2376 (88.3) | 772.8 (218.8, 2467.6) | 801.2 (202.7, 2495.3) | 708.6 (236.7, 2383.7) | 850.4 (293.3, 2439.6) | 0.546 | 0.376 |
| **Outcome, n (%)** |  |  |  |  |  |  |  |
| ESKD | 2692 (100) | 1157 (43.0) | 632 (42) | 406 (44) | 119 (44.7) | 0.539 | 0.495 |
| All-cause mortality | 2692 (100) | 799 (29.7) | 444 (29.5) | 268 (29.1) | 87 (32.7) | 0.509 | 0.509 |

^a^ Categorical variables are presented as frequency (%) and continuous variables are presented as median (IQR).

^b^ P-values are calculated by Wilcoxon rank sum test (Kruskal-Wallis test) for continuous variables and chi-square test for categorical variables. P-values for trend are calculated by Spearman’s correlation for continuous variables and by Cochran-Armitage trend test for categorical variables.

^c^ Baseline body mass index were the latest measurements that were obtained within -2 years to +2 years of the index date.

^d^ Baseline comorbidities and medication profiles that occurred within 1 year prior to the index date.

^e^ Baseline biochemical profiles were the latest measurements that were obtained within -1 year to +90 days of the index date.

CKD: chronic kidney disease, LDL-C: low-density lipoprotein cholesterol, HDL-C: high-density lipoprotein cholesterol, PCR: protein/creatinine ratio, ACR: albumin/creatinine ratio, ESKD: end-stage kidney disease.
