# Supplementary material for: Association of poorly controlled HbA1c with increased risk of progression to end-stage kidney disease and all-cause mortality in patients with diabetes and chronic kidney disease
Source: PLoS One. 2022 Sep 26;17(9):e0274605. doi: 10.1371/journal.pone.0274605 (PMC9512200; doi:10.1371/journal.pone.0274605)
Supplement: S2 Table — (DOCX) [file pone.0274605.s002.docx]

**S2 table.** Hazard ratios (95% confidence interval) of 30% decline of estimated glomerular filtration rate (eGFR), doubling serum creatinine, progression to end-stage kidney disease (ESKD), and all-cause mortality associated with baseline HbA_1c_ groups.

|  |  |  |  |  |  | **Model 1** | **Model 2** | **Model 3** |
| --- | --- | --- | --- | --- | --- | --- | --- | --- |
|  | N | cases | Person-years | Incidence^a^ | Crude HR  (95% CI) | Adjusted HR  (95% CI) | Adjusted HR  (95% CI) | Adjusted HR  (95% CI) |
| **30% decline of eGFR** ^b, c, d^ |  |  |  |  |  |  |  |  |
| Baseline HbA_1c_ < 7 % | 2125 | 835 | 6725.70 | 124.15 | 1.00 (Ref) | 1.00 (Ref) | 1.00 (Ref) | 1.00 (Ref) |
| Baseline HbA_1c_ 7-9 % | 1796 | 786 | 6128.28 | 128.26 | 1.08 (0.97, 1.20) | 1.08 (0.98, 1.21) | 1.07 (0.96, 1.19) | 1.15 (1.02, 1.29) |
| Baseline HbA_1c_ > 9 % | 619 | 259 | 2129.91 | 121.60 | 1.00 (0.86, 1.16) | 1.01 (0.87, 1.17) | 0.95 (0.81, 1.11) | 1.07 (0.91, 1.26) |
| *P* for trend |  |  |  |  | 0.5736 | 0.5020 | 0.8968 | 0.131 |
| **Doubling Serum creatinine** ^b, c, d^ |  |  |  |  |  |  |  |  |
| Baseline HbA1c < 7 % | 2125 | 716 | 9831.79 | 72.82 | 1.00 (Ref) | 1.00 (Ref) | 1.00 (Ref) | 1.00 (Ref) |
| Baseline HbA_1c_ 7-9 % | 1796 | 685 | 9052.38 | 75.67 | 1.11 (1.01, 1.22) | 1.10 (1.01, 1.21) | 1.02 (0.93, 1.12) | 1.06 (0.96, 1.16) |
| Baseline HbA_1c_ > 9 % | 619 | 260 | 3155.56 | 82.39 | 1.20 (1.06, 1.35) | 1.20 (1.06, 1.35) | 1.08 (0.95, 1.22) | 1.13 (0.99, 1.29) |
| *P* for trend |  |  |  |  | 0.0017 | 0.0021 | 0.2722 | 0.0599 |
| **Progression to ESKD** ^b, c, d^ |  |  |  |  |  |  |  |  |
| Baseline HbA_1c_ < 7 % | 2126 | 602 | 4626.14 | 130.13 | 1.00 (Ref) | 1.00 (Ref) | 1.00 (Ref) | 1.00 (Ref) |
| Baseline HbA_1c_ 7-9 % | 1798 | 428 | 4346.82 | 98.46 | 0.74 (0.65, 0.85) | 0.75 (0.66, 0.86) | 0.80 (0.69, 0.92) | 1.09 (0.94, 1.25) |
| Baseline HbA_1c_ > 9 % | 619 | 158 | 1485.67 | 106.35 | 0.69 (0.57, 0.83) | 0.70 (0.58, 0.84) | 0.73 (0.59, 0.90) | 1.16 (0.91, 1.46) |
| *P* for trend |  |  |  |  | < 0.001 | < 0.001 | < 0.001 | 0.167 |
| **All-cause mortality** ^c, d^ |  |  |  |  |  |  |  |  |
| Baseline HbA_1c_ < 7 % | 2126 | 810 | 8584.97 | 94.35 | 1.00 (Ref) | 1.00 (Ref) | 1.00 (Ref) | 1.00 (Ref) |
| Baseline HbA_1c_ 7-9 % | 1798 | 652 | 7941.58 | 82.10 | 0.91 (0.82, 1.01) | 0.92 (0.83, 1.02) | 0.93 (0.84, 1.04) | 1.06 (0.95, 1.18) |
| Baseline HbA_1c_ > 9 % | 619 | 236 | 2726.49 | 86.56 | 1.04 (0.90, 1.21) | 1.05 (0.91, 1.22) | 1.03 (0.88, 1.19) | 1.25 (1.07, 1.46) |
| *P* for trend |  |  |  |  | 0.781 | 0.926 | 0.821 | 0.009 |

^a^ Incidence = No. of incident 30% decline of eGFR, doubling serum creatinine, progression to ESKD, or mortality cases/ person-years*1000.

^b.^ Cox proportional hazards analysis with the competing risk of death by subdistribution hazard model was performed for the outcome of 30% decline of eGFR, doubling serum creatinine, and progression to ESKD.

^c^ Model 1: Adjusted for sex, body mass index, smoking status, alcohol consumption, education (Baseline HbA_1c_: n= 4543). Model 2: Further adjusted for systolic blood pressure, cardiovascular disease, primary etiologies of chronic kidney disease, baseline medication (contrast, nonsteroidal anti-inflammatory drugs, oral antidiabetic agents, insulin, angiotensin-converting enzyme inhibitors, angiotensin receptor blockers, diuretics, epoetin), triglyceride and low-density lipoprotein cholesterol. Model 3: Further adjusted for baseline hemoglobin, estimated glomerular filtration rate, and pooled urine protein/creatinine ratio.

^d^ Age was used as time scale.
